# Influence of Aerobic Power on Youth Players’ Tactical Behavior and Network Properties during Football Small-Sided Games

**DOI:** 10.3390/sports7030073

**Published:** 2019-03-25

**Authors:** Gibson Moreira Praça, Raphael Brito e Sousa, Pablo Juan Greco

**Affiliations:** Sports Department, Universidade Federal de Minas Gerais, Belo Horizonte 31270-901, Brazil; raphaelbes7@gmail.com (R.B.e.S.); grecopj@ufmg.br (P.J.G.)

**Keywords:** small-sided games, football, social network analysis, tactical behavior

## Abstract

(1) Background: This study aimed to compare the incidence of tactical principles, the percentage of successful tactical principles, and the network properties between higher and lower aerobic power in young football players during small-sided games. (2) Methods: Eighteen Under-17 Brazilian players were recruited. Firstly, they performed the Yo-Yo Intermittent Recovery Test Level 2, which was used to split them into two groups with higher and lower aerobic power. In the sequence, they played three vs three small-sided games within each group. The System of Tactical Assessment in Soccer was used to analyze the tactical behavior demonstrated by measuring the incidence of tactical principles and the percentage of successful principles, while the macro variables, density and clustering coefficient from social network analysis for team sports was used to analyze players’ interactions. (3) Results: No differences were reported for the incidence of tactical principles (*p* > 0.05, small or small-to-moderate effect sizes), the percentage of successful offensive principles (*p* = 0.122, small-to-moderate effect size), or the network variables (*p* > 0.05; small effect sizes). The lower aerobic power group demonstrated a higher percentage of successful defensive tactical principles (*p* = 0.043; small-to-moderate effect size). (4) Conclusions: We concluded that aerobic power has a limited impact on player behavior, indicating that players’ actions within a small-sided game are mostly constrained by other parameters.

## 1. Introduction

The training process in team sports must provide stimuli to a wide range of physical, tactical, and technical components [1]. For this reason, recent studies attempted to highlight the positive impact of small-sided games (SSG) to stimulate such components [2,3] in a game-based context, which raises the potential benefits of this training tool. Thus, many studies have investigated the manipulation of components of the SSG to elicit specific responses from players [4]. However, little attention has been paid to investigating the influence of individual players’ characteristics on the perceived demand, which limits the potential to adequately adjust training contents to the players’ specific needs. Specifically, studies show that the level of tactical knowledge [5], playing position [6], and age group [7] impact players’ responses to the same SSG, which requires caution from coaches using this training tool irrespective of the players’ characteristics. However, limited research is available about the influence of physical skills on high level youth soccer players’ responses during SSG.

In official matches, players usually cover distances higher than thirteen kilometers during ninety minutes [8]. Aerobic energy thus represents the priority energy source for players’ actions [9]. This highlights the importance of achieving high levels of aerobic power for good performance in matches [10]. The influence of aerobic power on the players’ physical performance has been established in the literature. For example, studies have reported a positive association between aerobic power and the total distance covered in official matches [11,12]. Furthermore, it has been suggested in the literature that fatigue mechanisms affect decision-making skills [13]. For this reason, it is expected that a higher level of aerobic power, which could reduce the effects of fatigue for the same training load, would result in better cognitive functioning during sports practice. From this, it is possible to expect that football players with higher levels of aerobic skills would present better tactical behavior in a game-based task like small-sided games. However, this hypothesis has not been previously tested in the literature.

Recently, the tactical behavior of football players in small-sided games has been investigated using new tools such the System of Tactical Assessment in Soccer (FUT-SAT) [14] and social network analysis (SNA) applied to team sports [15]. This fills an important gap, since the majority of studies regarding SSG have focused on physical responses. By using the FUT-SAT it is possible to quantify players’ incidence of core tactical principles in both offensive and defensive phases of the game. This system has been previously used to demonstrate that the individual characteristics of the players, when used as team composition criteria, can influence the tactical behavior [16]. Besides, the SNA has previously been used to investigate the influence of playing position on network centrality measures during SSGs [6]. Considering the previously discussed importance of aerobic power with regard to player performance during game-based tasks, it was expected that tactical performance would be constrained by the players’ aerobic skills because of its influence on fatigue during SSGs. Consequently, differences in the variables related to FUT-SAT and SNA were expected when comparing players with higher and lower aerobic skills in the same SSG. However, to the best of our knowledge, there is a gap in the literature regarding how groups with different aerobic power levels behave tactically in game-based tasks.

The assessment of players’ tactical performance in game-based soccer tasks has increased in recent years. Although previous instruments have been utilized (like the Game Performance Assessment Tool, discussed and adapted by Memmert and Harvey [17]), the high specificity of the variables presented in the FUT-SAT, linked to the wide range of variables included in the SNA, make these two important tools to be applied in studies of soccer SSG. Based on the rationale described above, this study had two aims: to compare the incidence of core tactical principles and the percentage of successful tactical principles between groups of higher and lower aerobic power, and to compare the network structure of teams composed of players with higher and lower aerobic power.

## 2. Materials and Methods

### 2.1. Participants

This study was approved by the local ethics committee of the Universidade Federal de Minas Gerais (CAE 51011915.9.0000.5149). Eighteen male Under-17 football athletes (16.4 ± 0.9 years, 62.9 ± 7.55 kg, 172.4 ± 6.76 cm) from a national class team youth academy (six defenders, six forwards, and six midfielders) were selected. They usually compete at the national level (Brazil) and had an average of 7 training sessions per week. All participants had at least five years of deliberate practice. Participants were included based on the head coach’s classification, in order to select players with a similar tactical–technical performance according to his subjective assessment. Players with injuries were excluded. The data collection took place in the middle of the competitive season (April and May). Prior to data collection, athletes and their legal guardians were provided with information about all of the research procedures, and written consent was obtained.

### 2.2. Procedures

Firstly, athletes performed the Yo-Yo Intermittent Recovery Test Level 2 (YYIRT2) [18]. The YYIRT2 has been reported to provide a low-cost and reliable measure of aerobic power [19]. During the test, the players completed 20 m shuttle runs, starting at 13 km/h and increasing the velocity with 10 s of active recovery between runs, until exhaustion. The test was considered finished when a player failed to reach the line on time on two consecutive occasions. The running pace was controlled by an audio signal, according to the recommendations in the literature [18].

After the YYIRT2, players were divided into two groups. Group 1 was composed of the nine players (three defenders, three midfielders and three forwards) who presented higher performance in the test, while Group 2 was composed of the nine players with the lower scores. After the division, the groups were significantly different in terms of the YYIRT2 score (*p* < 0.05). Within each group, three teams (A, B, and C) were formed, each with one defender, one midfielder and one forward. Teams played only small-sided games within each group, and all possible matches were realized once (AxB, AxC, and BxC). After all possible confrontations were observed within each group, the total of twelve bouts were analyzed. In this study, no direct sample size estimation was conducted, since the data came from a major study (in which the sample size was adequately estimated).

**Figure 1:**
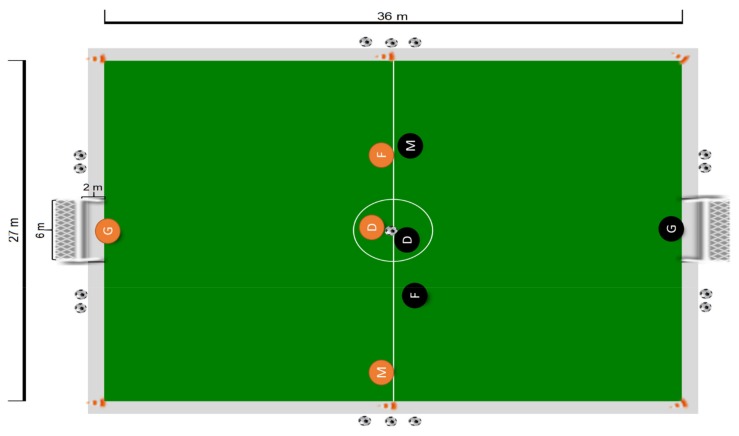
Representation of the experimental task. Notes: G: Goalkeeper; D: Defender; M: Midfielder; F: Forward.

In the sequence, teams performed 3 vs 3 (plus goalkeepers, not evaluated) SSGs. Every small-sided game comprised two bouts of four minutes with four minutes of passive recovery between games. The SSGs were played on a natural grass surface with a field size of 36 × 27 m, with all rules of the formal game in effect (including offside). Additional balls were placed around the field in order to allow the fast restarting of the game. Adapted 6 × 2 m goals were used. During each small-sided game, players were instructed to try to win the game by scoring more goals than the opponent team. Verbal encouragement was allowed, but no technical or tactical instructions were provided. Figure 1 represents the experimental task.

Each session started with a preparatory activity lasting 10 min, including actions with and without the ball. After this, the first bout was immediately started. During the rest period, consumption of water was allowed ad libitum. Each bout was recorded with a digital camera (JVC HD Everio GZ-HD520, JVCKENWOOD, Yokohama, Japan) placed 5 m above the field, which allowed a view of the whole field without changing its position. The video scenes were used for posterior analysis regarding the tactical principles and for social network analysis by experts.

### 2.3. Instruments

#### 2.3.1. System of Tactical Assessment in Soccer (FUT-SAT)

The FUT-SAT consists of observation of the incidence of 10 core tactical principles performed by athletes during a game [14]. Among these principles, 5 are related to the offensive phase (penetration, offensive coverage, width and length (with and without the ball), depth mobility, and offensive unit) and 5 are related to the defensive phase (delay, defensive coverage, balance (defensive and recovery), concentration, and defensive unit). Two trained observers analyzed the videos and counted the number of actions related to each principle performed by each athlete, with the use of the Soccer Analyzer^®^ software (version 1.0 alpha, by Eduardo Valgôde, Porto, Portugal). The software allows for the insertion of a field diagram on the video image and establishes the game center and ball line, references that are adopted for the definition of the tactical principles. The dependent variables were the incidence of each fundamental tactical principle and the percentage of successful offensive actions in both the offensive and defensive phase.

#### 2.3.2. Social Network Analysis

In this study, passes were used to establish connections, as recommended in the literature [15]. A successful pass occurred every time a player sent the ball to a teammate, who was able to keep possession of the ball without any significant interference in ball trajectory by an opponent player. An adjacency matrix was created for all SSGs, considering the successful passes performed by a player to a teammate. The software Social Network Visualizer (SocNetV 1.9 (C) 2005–2015 by Dimitris V. Kalamaras, Greece) was used to extract the SNA measures.

The general network properties of the SNA indicate the macro level of analysis, which includes the interactions between players from a collective perspective (e.g., considering the whole team). The macro analysis includes the density and the clustering coefficient. The density is the ratio between the observed (total) links and the maximum number of links (density values range from 0, or no density—lack of cooperation—to 1, or maximal cooperation). The clustering coefficient indicates the level of interconnectivity between close teammates (values range from 0, or no interconnectivity, to 1, maximal cooperation) [15].

### 2.4. Data Analysis

The assumptions of normality (Shapiro–Wilk) and homocedasticity (Levene) were first checked. In the sequence, an independent t-test was used to compare the incidence of tactical principles, the offensive performance, and the density between protocols. Because the assumption of normality was not assumed, a Mann–Whitney test was used to compare the data on penetration, width and length with the ball, depth mobility, offensive unity, delay, defensive coverage, defensive balance, recovery balance, concentration, and the clustering coefficient between protocols. Cohen’s d effect size were calculated and classified into small (d = 0.2), moderate (d = 0.5) or large (d = 0.8) [20]. 

To ensure the quality of the observations, 12.5% of the SSG bouts were reanalyzed by the same observers (intra-observer concordance) and by different observers (inter-observer concordance). These reanalyses occurred twenty-one days after the first observation. Kappa’s coefficient was calculated and revealed values higher than 0.8 for all variables. For this reason, the concordance was classified as “perfect” [21,22].

## 3. Results

Table 1 presents the data regarding the incidence of tactical principles and the percentage of successful tactical actions performed by each group. No differences were observed in the incidence of tactical principles (small and small-to-moderate effect sizes). However, group 2 presented a higher percentage of successful defensive principles (*p* = 0.043; small-to-moderate effect). In general, there was no impact of aerobic performance on tactical behavior during the 3 v 3 SSGs.

Table 2 presents the data on the general network measures. No differences were reported (small effect sizes). In summary, the players’ interactions were not affected by aerobic power.

## 4. Discussion

Investigations of aerobic power in soccer have been largely established in the literature. This has also been a topic of interest with regard to understanding player performance during game-based tasks (like SSGs), since recovery after a dynamic workout using large muscle groups with high-intensity effort (which leads to the production and accumulation of lactate) is dependent on aerobic sources of energy [23]. Better recovery between high intensity demands could increase players’ tactical performance due to better cognitive functioning. This has not been fully addressed in the available literature. Consequently, this study sought to expand the available literature regarding the role of aerobic power in players’ performance by comparing the incidence of core tactical principles, the percentage of successful tactical principles, and the network properties between groups of higher and lower aerobic power during small-sided games of football. It was hypothesized that aerobic power would influence players’ tactical behavior, with a better performance being demonstrated by the group with higher aerobic power. This hypothesis was not confirmed, since only difference in one dependent variable was reported.

Previous studies reported a reduction in physical performance throughout a series of four small-sided games of 4 min duration [24]. Since this reduction was mostly explained by fatigue effects, it was supposed that players with higher aerobic power would present a less prominent reduction in physical performance as a result of their superior ability to recover between high-intensity stimuli [9,23]. In this sense, higher aerobic power would allow players to quickly recover, for example, between repeated sprints during SSGs. This fact would increase the players’ cognitive performance, which is the basis of tactical actions [25]. A possible explanation for the current results is the SSG regimen adopted (only two series, with a stimulus/rest ratio of 1:1), which was probably not enough to significantly increase the physical demand to the point where players with higher aerobic power would present better performance. Considering this, it seems useful to adopt this regimen when new tactical principles are about to be taught, since even players with lower aerobic power could achieve satisfactory game comprehension and thus systematically increase their tactical skills. This is particularly useful when considering the impact of mental fatigue on players’ tactical performance, which is reported in the literature [26]. Future studies should address this question by investigating the influence of aerobic power on players’ tactical performance under different SSG regimens. In addition, playing position has been shown to impact players. 

The group with lower aerobic power also presented a higher defensive tactical performance, which is contrary to the initial hypothesis. A previous study showed that the team composition criteria affect players’ tactical performance [16]. Moreover, it has been shown that playing against superior teams increases players’ synchronization [27]. For these reasons, it could be suggested that, although tactical skills were not considered in the present study, the matches within the lower aerobic level group were probably more balanced in terms of the players’ tactical skills. This is also confirmed because players from different positions, who usually have different aerobic skills [28], were balanced in all teams (i.e., a defender, a midfielder, and a forward on each team). As a result, it could be expected that the investigated players faced a more challenging pedagogical environment within the lower aerobic group, which could have increased their positioning performance and explain the higher percentage of successful defensive principles. However, the experimental design of the current study was not suitable for confirming this hypothesis. For this reason, future studies in which both aerobic power and tactical skills are controlled are needed.

With regard to practical applications, the results of the current study may impact the potential of using the proposed training regimen (3 vs 3, two bouts of 4 min with 4 min of passive recovery) for tactical training in soccer. Specifically, the use of similar regimens is suggested when the aim of the sessions includes learning not-previously known tactical principles, which could lead to mental fatigue and reduce player performance [26]. Considering the current SSG regimen, it seems unnecessary to adopt a different tactical periodization for players with higher and lower aerobic skills within the same team, since in tasks with a low training load, the players’ aerobic capabilities did not constrain their tactical performance. Finally, considering the Long-Term Athlete Development Model (LTDM) [29], the players in the current study were between the “train to train” and the “train to competition” stages, in which an optimal window of trainability for aerobic skills is observed, due to maturational aspects [29]. Coaches should thus take into account the need to clearly control the training loads in order to optimize the expected gains and to provide a better training context for players’ development, avoiding overloading. 

In summary, this study showed a low impact of aerobic power on players’ tactical behavior and network properties. Future studies should investigate the influence of aerobic power on players’ tactical behavior in different SSG regimens and control for the influence of intervening variables such as tactical knowledge [16]. In addition, studies must address the multiple variables that can interfere with players’ physical skills (both aerobic and anaerobic) in a case–control design, in order to deeply understand the influence of aerobic power on players’ tactical behavior. This was considered a limitation of the present study. Finally, we recommend that future studies address issues regarding the tactical aspects of different football SSGs, since this remains lacking in the literature in comparison to physical aspects. To achieve this, both the FUT-SAT and the SNA are expected to provide valuable information for coaches to better adjust SSG formats to their pedagogical aims. 

## Figures and Tables

**Table 1:** Mean (standard deviation) of the incidence of tactical principles and percentage of successful tactical actions performed by the groups.

Variable	Group 1	Group 2	*p*-Value	Effect Size
Offensive Principles				
Penetration	4.58 (1.92)	4.00 (2.29)	0.200	0.27
Offensive coverage	5.67 (3.06)	5.89 (2.35)	0.157	0.08
Width and length without ball	10.08 (5.23)	11.25 (4.81)	0.895	0.23
Width and length with ball	1.28 (1.23)	1.61 (1.49)	0.361	0.24
Depth mobility	2.17 (2.46)	1.86 (1.71)	0.945	0.14
Offensive unity	9.56 (4.38)	8.14 (4.58)	0.212	0.31
Defensive Principles				
Delay	6.86 (3.12)	6.81 (3.22)	0.959	0.01
Defensive coverage	1.94 (1.98)	2.19 (2.16)	0.722	0.12
Defensive balance	4.72 (3.48)	4.81 (2.96)	0.679	0.02
Recovery balance	3.58 (2.10)	2.72 (2.09)	0.149	0.41
Concentration	3.31 (2.43)	4.42 (3.04)	0.108	0.40
Defensive unity	15.86 (5.30)	14.97 (5.93)	0.428	0.15
Successful Tactical Principles				
Offensive	0.720 (0.097)	0.758 (0.111)	0.122	0.39
Defensive	0.506 (0.220)	0.611 (0.211)	0.043 *	0.48

* Significant difference.

**Table 2:** Mean (standard deviation) of the team network structure observed in each group.

Groups	Density	Clustering Coefficient
Group 1	0.874 (0.069)	0.825 (0.140)
Group 2	0.888 (0.100)	0.812 (0.178)
*p*-value	0.783	0.885
Effect Size	0.16	0.08
